# Proteoglycan expression correlates with the phenotype of malignant and non-malignant EBV-positive B-cell lines

**DOI:** 10.18632/oncotarget.5984

**Published:** 2015-10-16

**Authors:** Alexandra Y. Tsidulko, Liudmila Matskova, Lidiia A. Astakhova, Ingemar Ernberg, Elvira V. Grigorieva

**Affiliations:** ^1^ Institute of Molecular Biology and Biophysics, Novosibirsk, Russia; ^2^ Department of Microbiology, Tumor and Cell Biology (MTC), Karolinska Institute, Stockholm, Sweden; ^3^ Kemerovo Institute of Food Science and Technology, Kemerovo, Russia

**Keywords:** Epstein-Barr virus, B cells, lymphoma development, proteoglycan, heparan sulfate biosynthesis

## Abstract

The involvement of proteoglycans (PGs) in EBV-host interactions and lymphomagenesis remains poorly investigated. In this study, expression of major proteoglycans (syndecan-1, glypican-1, perlecan, versican, brevican, aggrecan, NG2, serglycin, decorin, biglycan, lumican, CD44), heparan sulphate (HS) metabolic system (EXT1/2, NDST1/2, GLCE, HS2ST1, HS3ST1/2, HS6ST1/2, SULF1/2, HPSE) and extracellular matrix (ECM) components (collagen 1A1, fibronectin, elastin) in primary B cells and EBV carrying cell lines with different phenotypes, patterns of EBV-host cell interaction and viral latency stages (type I-III) was investigated. Primary B cells expressed a wide repertoire of PGs (dominated by serglycin and CD44) and ECM components. Lymphoblastoid EBV+ B cell lines (LCLs) showed specific PG expression with down-regulation of CD44 and ECM components and up-regulation of serglycin and perlecan/HSPG2. For Burkitt's lymphoma cells (BL), serglycin was down-regulated in BL type III cells and perlecan in type I BL cells. The biosynthetic machinery for HS was active in all cell lines, with some tendency to be down-regulated in BL cells. 5′-aza-dC and/or Trichostatin A resulted in transcriptional upregulation of the genes, suggesting that low expression of ECM components, proteoglycan core proteins and HS biosynthetic system is due to epigenetic suppression in type I cells. Taken together, our data show that proteoglycans are expressed in primary B lymphocytes whereas they are not or only partly expressed in EBV-carrying cell lines, depending on their latency type program.

## INTRODUCTION

Proteoglycans (PGs) are complex macromolecules composed of a core protein and covalently linked polysaccharide chain(s) which play a critical role in cell-cell and cell-matrix interactions. Disruptions of such interactions might affect B cell interaction with surrounding stroma and may thus perturb the cell phenotypes. The possible role of PGs has so far been largely neglected, both during B-cell differentiation and as a factor in EBV-driven lymphomagenesis [1, 2].

It has been shown that PGs and their polysaccharide chains (especially heparan sulfates, HS) play an important role in the immune system participating in leukocyte development and migration, immune activation and inflammatory processes as well as in lymphoma development [3]. Tightly regulated HSPGs expression is a requirement for normal B cell maturation, differentiation and function [4] and the conformation of their HS polysaccharide chains is crucial for recruitment of factors that control plasma cell survival [5]. Correct modification of HS by the HS-modifying enzyme glucuronyl C5-epimerase (Glce), which controls HS chain flexibility, is required for proper lymphoid organ development [6]. Exogenous extracellular heparin inhibits BL cell proliferation while internalized heparin promotes it [7], and treatment of B cells with chondroitin sulphate (CS) activates B cells *in vitro* and induces HSPG CD138/syndecan-1 expression, affecting humoral immune response in mice [8].

Although a functional role of proteoglycans in normal B cell physiology and malignant transformation has been documented, controversies remain on PGs expression patterns in different immune cell types.

The CSPG serglycin is identified as a dominant PG in immune cells with an important functional role in immune system processes and inflammation [9, 10]. It is a major CSPG expressed by primary lymphocytes, although lymphoid cell lines express both serglycin and one or more types of cell surface proteoglycans of the syndecan/glypican families, displaying a presence of HS at their cell surface [11]. Syndecan-1 (CD138), a transmembrane HSPG, functions as a matrix receptor by binding cells to interstitial collagens, fibronectin, and thrombospondin. In bone marrow, syndecan is expressed only on precursor B cells. Syndecan 1) is lost immediately before maturation and release of B lymphocytes into the circulation, 2) is absent on circulating and peripheral B lymphocytes, and 3) is re-expressed upon their differentiation into immobilized plasma cells. Thus, syndecan mediates B cell stage-specific adhesion [12, 13]. Syndecan is expressed in chronic lymphocytic leukaemia B-CLL, both in tissue environment and in circulation [14, 15]. Syndecan expression is not detected in normal and malignant T cells [16]. Polysaccharide chains of syndecan-1 may contribute to homotypic adhesion and take part in the regulation of cell proliferation and active cell death in HT58 lymphoma cells [17].

Besides a functional role of PGs in the immune system, they are shown to be involved in virus-host cell interactions [18–20], including enterovirus 71 (EV71) [21], human immunodeficiency virus (HIV-1) [22], foamy virus (FV) [23], herpes virus 8 (HHV-8) [24], herpes simplex virus type-1 (HSV-1) [25, 26]. Some PGs have also been studied in EBV-associated cancers and premalignant conditions: chondroitinsulfate proteoglycan CD44 is detected in EBV-associated NPC [27–29] and EBV-related gastric carcinoma [30]; syndecan-1 (CD138) has been suggested to play a role in EBV-related PTLD [31].

PGs might also be involved in EBV infection of human lymphoid cells and affect EBV-host cell interaction and even lymphoma development.

Most investigated is CD44, the receptor for hyaluronic acid (HA), implicated in enhanced lymphoid tumor growth and dissemination. Although no changes in CD44 expression levels are shown during B cell activation by experimental EBV infection [32], it seems to be differentially associated with EBV-transformed lymphoblastoid cell lines and Burkitt's lymphoma cells biology. EBV-transformed LCLs abundantly express CD44, which is absent or minimally expressed in EBV-positive or EBV-negative BL cell lines [33]. However, the treatment EBV+ BL cells with B cell mitogen phorbol 12-myristate 13-acetate (PMA) or cytokine IL-4 enhances expression of an isoform H of CD44 and induces strong HA recognition in the cells. The ability to recognize HA was not observed in B-LCL cells stimulated with either PMA or IL-4 suggesting selective inactivation of molecular pathways that regulate CD44 expression and CD44-mediated HA binding in LCL cells [34]. Introduction of EBV latent membrane protein I (LMP1) gene into BL cells induces expression of CD44 on the cell surface suggesting that expression of LMP1 may regulate expression of CD44 and play a role in the behavior of EBV-based lymphomas [35].

An involvement of serglycin and syndecan-1/CD138 in EBV-host interactions has also been reported. Experimental infection of terminally differentiated tumor derived B cells (multiple myeloma, MM) with EBV virus *in vitro* results in down-regulation of syndecan-1/CD138 expression [12]. EBV infection of BL cells *in vitro* significantly up-regulates expression of nine genes including those encoding serglycin core protein and CD44 [36]. The data suggest a possible involvement of PGs in EBV-driven lymphangiogenesis, but the matter was not thoroughly investigated. The full spectrum of proteoglycans involved and possible molecular mechanisms of their involvement remain unclear.

In this study, we investigate the expression of proteoglycans, the metabolic system for HS chains biosynthesis, modification and degradation, and some key extracellular matrix components (collagen 1A1, fibronectin, laminin) in EBV infected B cell lines.

## RESULTS

### PG expression in human lymphoid cells is associated with EBV infection

A panel of EBV+ Burkitt's lymphoma cell lines with different EBV latency stages (type III: Raji, Daudi, Mutu III cl 99; type I: Rael, Akata, Mutu I cl148) and lymphoblastoid B cell lines generated by EBV transformation of normal human B cells *in vitro* (CBMI-Ral-STO, CBC-JK2-STO, Nad20) were used (Table 1). Altogether they represent a unique experimental cell line model to investigate molecular mechanisms of EBV effects on malignant transformation of human B cells [37].

**Table 1:** Cell lines characterization

Cell line	EBV latency	Malignant	Clumps	Virus source	*In vitro* transfection	Cell source
CBM1-Ral-STO	III	−	+++	Rael	+	Human B-cell (cord blood)
CBC-JK2-STO	III	−	+++	Jijoye	+	Human B-cell (cord blood)
Nad20	III	−	+++	B95–8	+	Human B-cell (Adult Blood)
Rael	I	+	−	Tumor	−	Burkitt Lymphoma
Akata	I	+	−	Tumor	−	Burkitt Lymphoma
Mutu I cl.148	I	+	−	Tumor	−	Burkitt Lymphoma
Mutu III cl.99	III	+	++	Tumor	−	Burkitt Lymphoma
Raji	II-III	+	+	Tumor	−	Burkitt Lymphoma
Daudi	II-III	+	+	Tumor	−	Burkitt Lymphoma

**Figure 1:**
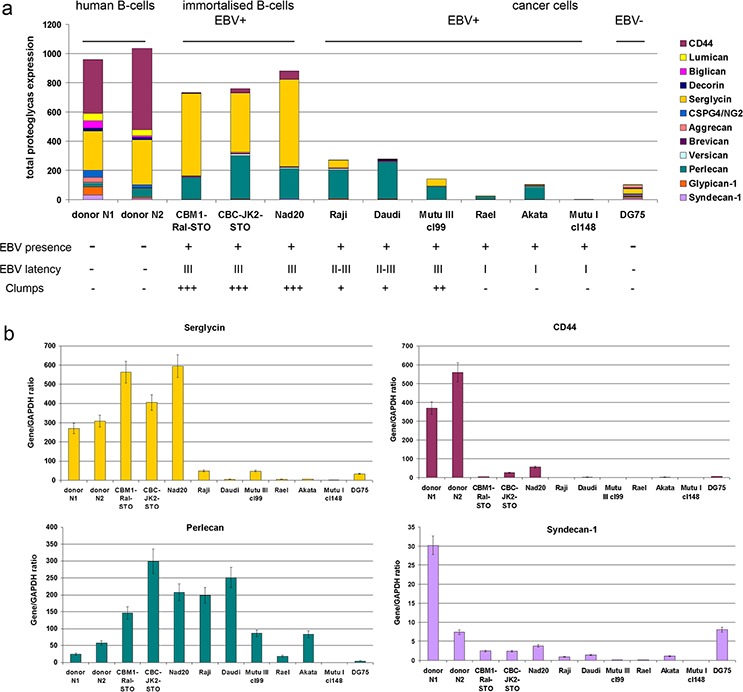
Proteoglycan expression in different normal and lymphoid cell lines **a.** Quantitative Real-Time RT-PCR. Intensity of the amplified DNA fragments for individual PGs normalized to that of GAPDH. Stacked column compares the contribution of each proteoglycan to the total PGs biosynthetic activity of the cells. **b.** Individual proteoglycans expression in the cell lines. The graph shows the mean expression levels from triplicate experiments (± SD) (OriginPro 8.1).

Primary B-cells and EBV- lymphoma cell line (DG75) were used as a control. Expression of main PGs (syndecan-1, glypican-1, perlecan, versican, brevican, aggrecan, NG2, serglycin, decorin, biglycan, lumican and facultative chondroitinsulfate proteoglycan CD44) in the cell lines was studied by real-time RT-PCR analysis (Figure 1, Supplementary Table S1).

The overall expression of a pool of different PG core proteins is presented as composed of the sum of expression levels for all the proteoglycans under study (Figure 1a). The main PGs expressed by normal human B cells were CSPGs CD44 and serglycin, whereas EBV-immortalized B cells (LCL) expressed mainly serglycin and the HSPG perlecan. EBV+ BL cells showed moderate expression levels of perlecan only, if any (Figure 1a). Immortalization of the cells (LCL) have resulted in significant down-regulation of CD44 and 4–5-fold increase of perlecan expression shifting the overall CSPG:HSPG ratio towards HSPG.

EBV infected LCL and BL cell lines thus showed a different profile of PG expression. Overall transcriptional activity of PG-coding genes in LCL cells was not significantly different although the pattern of PG expression was changed, dominated by the significant down-regulation of CD44 and up-regulation of serglycin and perlecan (Figure 1a, 1b). BL cell lines demonstrated impaired potential for PG expression. Perlecan was the dominant PG in EBV+ BL cells independent of the different EBV latency profiles (Figure 1a). Interestingly, the Akata cell line was most similar to EBV latency II-III cell lines in this respect.

**Figure 2:**
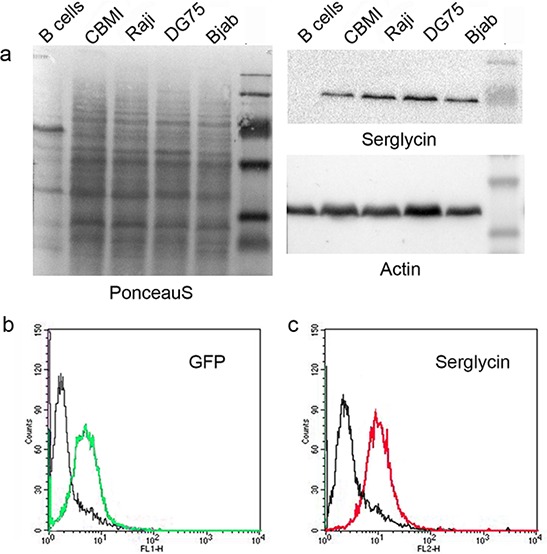
Serglycin expression in different normal and lymphoid cell lines **a.** Western blot analysis. bc. Serglycin expression at the cell surface of Raji cells upon super-infection with recombinant GFP-coding EBV virus. **b.** EBV infected Raji cells (green - GFP- EBV). **c.** Serglycin expression on GFP-EBV infected Raji cells (red).

As to individual PGs, EBV infection correlated to a significant (five to ten times) down-regulation of CD44 expression in EBV+ LCL cells (CBMI-Ral-STO, CBC-JK2-STO, Nad20). Neither EBV (+) nor (−) BL cell lines expressed CD44 regardless of the EBV latency stage (Figure 1a, 1b). Serglycin was expressed in normal B cells and LCLs only, whereas none of the BLs expressed this PG at a significant level (Figure 1a, 1b). Interestingly, some serglycin protein could still be detected at the protein level in EBV+ latency III BL cell lines (Figure 2a). Super-infection of the BL cell line Raji resulted in an increase of serglycin on the cell surface suggesting an involvement of EBV-infection in transcriptional regulation of serglycin (Figure 2b).

Along with the increased serglycin expression in LCLs, significant up-regulation of perlecan expression was detected (CBMI-Ral-STO, CBC-JK2-STO, Nad20) (Figure 1b, Supplementary Table S1). This together with increased expression levels of perlecan in BL latency II-III cells and its down-regulation (or disappearance) in the EBV+ latency I BL cells suggest that the perlecan expression could be associated with the EBV latency program of the host cells. In all cell types (B cells, LCL, BL cells) perlecan expression correlated with the adhesive capacity of the cells, which relate to the possible role of the PGs in cell-cell adhesion, however which is also dependent on ICAM-1. The results show that the type of EBV latency program (I or III) is strongly correlated with a certain PG expression profile in LCLs and BL cells.

### Different molecular mechanisms drive proteoglycans down-regulation at the different EBV latency stages

**Figure 3:**
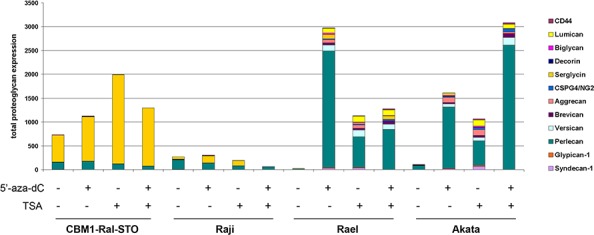
Changes in the expression of different PGs in LCL and BL cells in different latency stages after 5-aza-dC and TSA treatment The intensity of the amplified DNA fragments was normalized to that of *GAPDH*. Stacked column shows the added sum of PGs measured.

To reveal possible molecular mechanism of PG suppression in the cell lines, we experimentally modulated the EBV latency. It is known that the DNA demethylating agent 5′-deoxyazacytidine (5′-aza-dC) and Trihostatin A (TSA) – a chromatin structure modulator - are able to activate expression of EBNA2–6 and the LMP genes in latency I cells switching them towards latency II-III [38, 39]. To study the possible changes in PGs expression upon this switch, type I cells Rael and Akata as well as LCL cells (CBMI-Ral-STO) and BL latency III cells (Raji) were treated with 5′-aza-dC, TSA or both together and subjected to real-time RT-PCR analysis of the PG expression (Figure 3).

No significant changes in PG expression were seen upon aza/TSA treatments in LCLs (CBMI-Ral-STO) or BL of type II-III cells (Raji), although these treatments were capable to increase serglycin expression levels 2,5-fold in LCLs. In contrast to that, type I BLs were very sensitive to the aza/TSA treatment(s) showing activation of different PGs with a dominating up-regulation of perlecan/HSPG2. Different EBV latency I cell lines (Rael and Akata) showed specific sensitivity to the different drugs (5′-aza-dC was more effective in regulation of Rael cells, and TSA was more effective in Akata cells).

The results show that the loss of serglycin expression is a characteristic sign of EBV-positive BLs independently of the latency stages. The overall activation of PG expression upon 5′-aza-dC/TSA treatment in BL latency I but not latency III cells suggests an epigenetic regulation of PG expression. Different molecular mechanisms seem to control the PG down-regulation in EBV+ BL cells in different latency stages.

### Transcriptional activity of the HS biosynthetic machinery in normal and lymphoid cells in different EBV latency stages

Because of the complex protein-polysaccharide nature of proteoglycans, changes could occur both at the protein level (expression of the core proteins of the PGs) or at the polysaccharide (glycosaminoglycan, GAG) level. Biosynthesis and post-synthetic modification of the GAG chains are governed by a complex system of specific enzymes through the un-template mechanism. Their proper expression and ordered activity is of vital importance for the biosynthesis of functionally active PGs.

**Figure 4:**
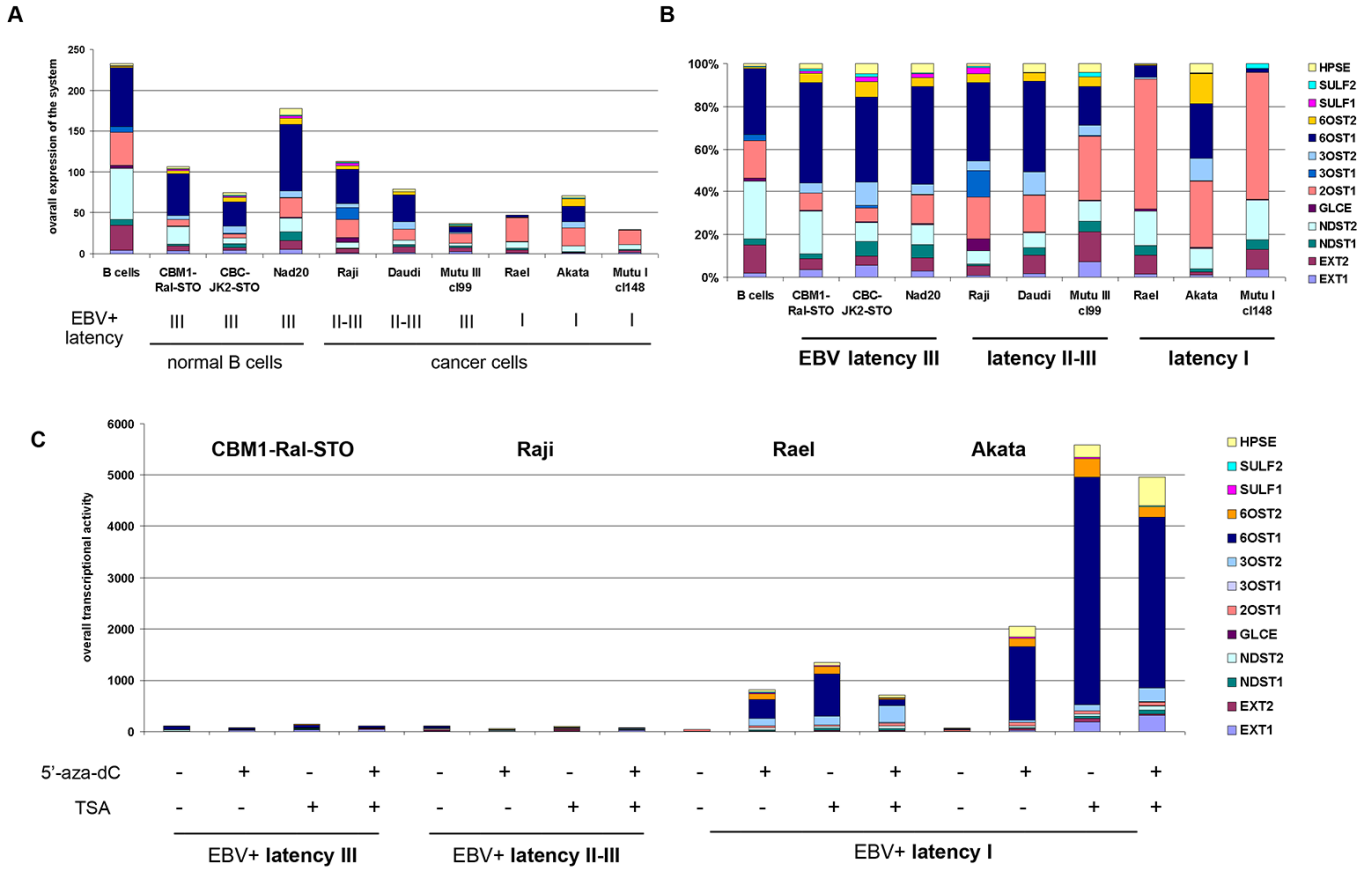
Expression of the HS biosynthetic system in primary B cells, LCLs and BL cells in different latency stages **a.** Quantitative Real-Time PCR. Intensity of the amplified DNA fragments for individual enzymes were normalized to that of *GAPDH*. Stacked column shows a comparison of the contribution of each enzyme to the total transcriptional activity of the HS biosynthetic system in each cell type. **b.** Qualitative structure of the expression of HS biosynthetic system. Expression rates for the individual genes were calculated as percentage of the total transcriptional activity of the system, set to 100%. **c.** Effects of 5′-aza-dC and TSA treatments on HS biosynthetic system transcriptional activity in LCL and BL cells in different latency stages.

To analyse the transcriptional activity of heparan sulfate (HS) biosynthetic system, we determined expression levels of the key HS biosynthetic enzyme genes in B-cells, LCLs and BL cells in different latency stages (Table 1) using real-time RT-PCR analysis (Figure 4).

According to the RT-PCR data, overall transcriptional activity of HS-metabolic system was not drastically different between the cell lines (Figure 4a). However, differential expression of individual HS biosynthesis-involved genes resulted in cell type-specific transcriptional patterns of HS biosynthetic machinery (Figure 4b). LCLs and BL type of II-III cells had similar overall transcriptional patterns and expression levels for these genes, with very high expression of 6OST1/HS6ST1 gene (6-O-sulfotransferase, responsible for the modification of HS polysaccharide chain with sulfate group at 6-position). In contrast, BL type I cells demonstrated moderately impaired overall transcriptional activity of the machinery and specific expression patterns. 6OST1/HS6ST1 expression was significantly inhibited in the cells along with an increased expression of 2OST1/HS2ST1 (2-O-sulfotransferase, which is responsible for the modification of HS polysaccharide chain with sulfate group at 2-position – a more rare modification).

Interestingly, the 5′-aza-dC/TSA treatment specifically affected the transcriptional activity of HS biosynthesis-genes in BL latency I cells (Rael, Akata) restoring high expression levels of 6OST1/HS6ST1 and shifting their overall transcriptional patterns to that of BL latency II-III cells. This suggests an epigenetic inactivation of the HS biosynthetic system in the BL type I cells (Figure 4c). At the same time, no evident effect of the 5′-aza-dC/TSA treatment(s) could be seen in LCL or BL type of II-III cells.

The results show that HS biosynthetic machinery is transcriptionally active in all cell lines under study, although some qualitative differences were detected mainly due to the epigenetic down-regulation of HS biosynthesis-involved genes (especially 6OST1/HS6ST1) in BL latency I cells.

### Expression of extracellular matrix components in EBV+ and EBV- lymphoid cells

**Figure 5:**
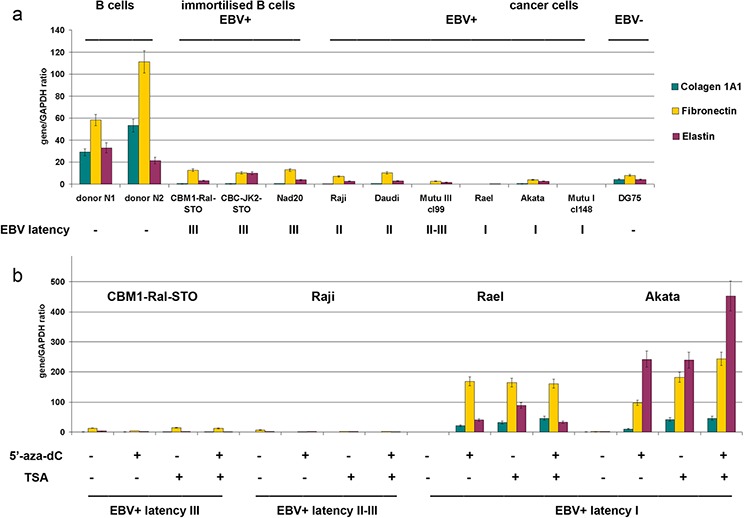
Expression of extracellular matrix components in primary B cells, LCLs and BL cells in different latency stages **a.** Quantitative Real-Time Q-PCR. Intensity of the amplified DNA fragments normalized to that of *GAPDH*. Bars represent the mean ± SD from triplicate experiments (OriginPro 8.1). **b.** Effects of 5′-aza-dC and TSA treatment on the HS biosynthetic system transcriptional activity in LCL and BL cells in different latency stages.

A similar approach was used also to study the expression of main components of the extracellular matrix (ECM) (collagen 1A1, fibronectin, elastin) (Figure 5).

According to the RT-PCR data, B-cells expressed collagen 1A1, fibronectin and elastin while LCL and BL cells did so only at a very low level (Figure 5a). 5′-aza-dC/TSA treatment(s) was capable to activate these genes only in BL type of I cells but not in BL type of II-III cells suggesting different molecular mechanisms of gene inactivation (Figure 5b).

## DISCUSSION

We show that normal primary B lymphocytes express a broad repertoire of PG core proteins, with CD44 and serglycin as major PGs. Our data are in line with previously published results showing that CD44 [32], serglycin [9–11] and syndecan-1/CD138 [15, 16] are expressed in B lymphocytes. In addition we demonstrate for the first time that B cells express additional proteoglycans (perlecan, glypican-1, CSPG4/NG2, decorin, biglycan, lumican) and a wide variety of PGs is consistently altered in EBV-carrying LCLs compared to primary B lymphocytes.

Interestingly, B cells also express extracellular components like collagen 1A1, fibronectin and elastin. This supports published data that B cells from bone marrow of healthy donors contribute with fibronectin and collagen 1A1 production to the cellular microenvironment [40] and that they are able to activate collagen production by fibroblasts [41, 42]. It has been shown that extracellular fibronectin and collagen 1A modulate adhesion of B cells to ECM, which in turn can affect B cell differentiation [43], and contribute to the development of fibrosis and systemic sclerosis [41, 42]. Short-term exposure to collagen is sufficient to significantly decrease the survival of DG75 EBV-negative Burkitt lymphoma cells or L428 Hodgkin lymphoma cells via up-regulation of DDR1 [44].

Malignant BL cells showed a pronounced inhibition of PG- and ECM components-genes compared to normal B-cells, with perlecan as the major PG in the cells (Figure 1, 5). Earlier, lack of CD44-expression in EBV-positive or EBV-negative BL cell lines [33], up-regulation of serglycin and CD44 in EBV+ BL cells [36], down-regulation of syndecan-1/CD138 in EBV-infected multiple myeloma cells [15] and fibronectin and collagen in multiple myeloma patients [40] have already been reported [33].

The low expression levels of PGs and ECM in BL cells correlated with the different EBV latency program [45]. While PG inhibition in BL latency I cells is ensured by epigenetic silencing of the corresponding genes and could be reversed by exposure to 5′-azacytidin and/or Trichostatin A, LCLs and BL latency III cells did not respond to this treatment. These data fit the well-established overall epigenetic profile of these cell lines with epigenetic mechanisms involved in the control of EBV-coding genes (EBNA2–6, LMP1,2) [46, 47] as well as hypermethylation of the host cell genome [48] in EBV latency I cells. EBV-associated cancers are characterized by conspicuous hypermethylation of both the viral genome and cellular genes, including tumor suppressor genes [49, 50]. LCLs, in contrast, show a pattern of relative hypomethylation of the viral and cellular genomes. It has even been suggested that EBV infection may contribute to tumor development by imposing a “methylator” phenotype [51, 52]. Thus, proteoglycans and ECM molecules seem to belong to the large set of genes inactivated by epigenetic mechanism in EBV+ tumor derived cells. Upregulation of PGs and ECM components in B cells by 5-aza-dC and TSA could affect the immune response to these cells *in vivo*.

It is important to note that we focused – for technical reasons - on the PG core proteins without considering their glycosylation with GAG chains. However, transcriptional activity and expression pattern of HS biosynthesis-involved genes show correlation with the PG core protein expression and is similarly controlled by epigenetic regulation (Table 2). These data support our hypothesis that a molecular mechanism coordinating the transcriptional activities of the systems to optimize the production of these complex polysaccharide-protein molecules exists [53].

**Table 2:** Summary of the obtained results

Cell line	EBV latency	Proteoglycans	HS biosynthetic enzymes	ECM molecules
primary B cells	-	serglycin, CD44	EXT2, NDST2, 2OST1, 6OST1	fibronectin, collagen1A1, elastin
CBM1-Ral-STO	III	serglycin, perlecan	NDST2, 6OST1	fibronectin
CBC-JK2-STO	III	serglycin, perlecan	6OST1	fibronectin
Nad20	III	serglycin, perlecan	6OST1	fibronectin
Mutu III cl.99	II-III	perlecan	2OST1	-
Raji	II	perlecan	2OST1, 3OST1, 6OST1	-
Daudi	II	perlecan	2OST1, 6OST1	-
Akata	I	perlecan	2OST1, 6OST1	-
Rael	I	-	2OST1	-
Mutu I cl.148	I	-	2OST1	-

The genes with the highest relative expression levels are shown for each cell line. dash–gene(s) are expressed at very low level.

Taken together, our results put focus on the important contribution of proteoglycans in B cell interaction with its microenvironment during differentiation and emphasizes the need to explore further the potential role of PGs and ECM molecules in lymphoma development.

## MATERIALS AND METHODS

### Cell lines and cell culture

The CBMI-Ral-STO, CBC-JK2-STO, Nad20, Raji, Daudi, Mutu III cl 99, Rael, Akata, Mutu I cl148 and DG75 human cell lines with different EBV latency stages were obtained from MTC (Karolinska Institute, Sweden). All cell lines were maintained in RPMI medium supplemented with 2 mM L-glutamine, 100 units/ml penicillin, 100 μg/ml streptomycin, and 10% fetal bovine serum at 37°C in a humidified 5% CO_2_ incubator. For analysis, cells were harvested by centrifugation at 1000 rpm for 5 min at RT. Cell lines characterization is shown in Table 1.

### 5-aza-dC/TSA treatment

Treatment with 5′-deoxyazacytidine (5-aza-dC, 4 μg/ml) or Trichostatin A (TSA, 200 ng/ml) was performed by incubating the cells with the drugs for 72 h or 24 h, respectively. For combined treatment, the cells were incubated with 5-aza-dC (4 μg/ml) for 48 h followed by TSA (200 ng/ml) for an additional 24 h.

### Human primary B cell isolation

B cells were isolated from PBMCs of healthy blood donors (Blood Transfusion Center Solna, Stockholm, Sweden) according [54]. Briefly, B cells were isolated either through negative selection (B Cell Isolation Kit II; Miltenyi Biotec) or by positive selection using biotinylated anti-IgD Ab (Southern Biotech) and Anti-Biotin MicroBeads (Miltenyi Biotec). The purity and B cell composition of each donor were assessed by flow cytometry, staining for CD19-allophycocyanin (HIB19; BD), IgD-FITC (IA6–2; BD), CD38-PECy (HB7; BD), CD27-PE (M-T271; BD), and DAPI (5.7 μM; Sigma-Aldrich) using an LSR II or Fortessa (BD) and analyzed using FlowJo software (TreeStar).

### RT-PCR analysis

Total RNA was extracted from the cells using the Qiagen RNeasy total RNA purification System (Qiagen) according to the manufacturer's instructions. cDNA was synthesized from 1 to 2 μg of total RNA using a First Strand cDNA Synthesis kit (Fermentas, Hanover, MD, USA) and 1/10th of the product was subjected to PCR analysis. Quantitative real-time RT-PCR (qRT-PCR) was performed using the ABI PRISM 7500 Sequence Detector (AppliedBiosystems, USA) and the Maxima SYBR Green/RO master mix (Thermo Scientific) under the following conditions: 50°C for 2 min, 95°C for 10 min, followed by 40 cycles at 95°C for 15 s and 60°C for 1 min. The total reaction volume was 25 μl. *GAPDH* was used as the housekeeping gene. The PCR primers are listed in [55].

### Western blotting

Cells were lysed with RIPA-buffer containing Protease Inhibitor Cocktail (Sigma), incubated for 30 min on ice, centrifuged for 15 min at 13000g. The protein concentration was quantified using Bradford Bio-Rad Protein Assay (BioRad). Total proteins (15 μg) were treated with NuPAGE LDS Sample Buffer (Life Technologies) containing 10% β-mercaptoethanol for 5 min at 96°C, resolved in 10% SDS-PAGE gels and transferred to nitrocellulose membranes Amersham Protran 0.45 NC (Amersham). The membranes were blocked with 5% milk in PBST (0,1%) for 1 h and incubated with primary antibodies [mouse anti-serglycin monoclonal (Santa Cruz, 1:200) or mouse anti-β-Actin monoclonal (Santa Cruz), 1:500] overnight at 4°C followed by secondary goat anti-mouse IgG (H+L)-HRP peroxidase-conjugated antibodies (BioRad) for 2 h at RT. Proteins were detected with an Amersham ECL Western Blotting Detection Reagent (Amersham) according to the manufacturer's instructions.

### EBV infection of human BL cells

EBV-containing conditioned medium was collected from a gastric carcinoma AGS cell line carrying recombinant EBV-GFP (40 h incubation, monolayer). For *in vitro* infection, 10^6^ Raji cells were pelleted by centrifugation at 1000 prm for 5 min at RT, resuspeded in 2 ml virus–containing supernatant with 8 mkg/ml polybrene and incubated on 6-well plate in a humidified incubator at 37°C and 5% CO_2_ for 90 h with shaking every 24 h. For analysis, cells were harvested by centrifugation at 1000 rpm for 5 min at RT.

### Immunostaining and FACS analysis

Cells were washed with PBS, resuspended in 1ml PBS, 1ml 70% ethanol was added and the cells were incubated for 30 min at 4°C. Then, the cells were washed with PBS, resuspended in mouse anti-serglycin monoclonal primary antibodies in PBS (Santa-Cruz; 1:50) and incubated for 30 min at RT. Staining patterns were visualised with Alexa Fluor^®^ 488-conjugated Goat anti-Mouse IgG (H+L) Secondary Antibodies (Life Technologies, 1:100, 30 min at RT) and analysed using FACSCalibur flow cytometer (BD Biosciences) with CellQuest Pro program.

### Statistical analysis

Statistical analyses were performed using a computer program ORIGIN Pro 8.1; *a* value of *p* < 0.05 was considered as statistically significant. Data are expressed as means ± SEM.

## SUPPLEMENTARY TABLE



## Abbreviations

PG: proteoglycan
HS: heparan sulfate
CS: chondroitin sulfate
BL: Burkitt's lymphoma
EBV: Epstein-Barr virus
LCL: EBV-positive lymphoblastoid B cell line

## References

[1] Bornkamm GW (2009). Epstein-Barr virus and the pathogenesis of Burkitt's lymphoma: more questions than answers. Int J Cancer.

[2] Rowe M, Fitzsimmons L, Bell AI (2014). Epstein-Barr virus and Burkitt lymphoma. Chin J Cancer.

[3] Simon Davis DA, Parish CR (2013). Heparan sulfate: a ubiquitous glycosaminoglycan with multiple roles in immunity. Front Immunol.

[4] Reijmers RM, Spaargaren M, Pals ST (2013). Heparan sulfate proteoglycans in the control of B cell development and the pathogenesis of multiple myeloma. FEBS J.

[5] Reijmers RM, Groen RW, Kuil A, Weijer K, Kimberley FC, Medema JP, van Kuppevelt TH, Li JP, Spaargaren M, Pals ST (2011). Disruption of heparan sulfate proteoglycan conformation perturbs B-cell maturation and APRIL-mediated plasma cell survival. Blood.

[6] Reijmers RM, Vondenhoff MF, Roozendaal R, Kuil A, Li JP, Spaargaren M, Pals ST, Mebius RE (2010). Impaired lymphoid organ development in mice lacking the heparan sulfate modifying enzyme glucuronyl C5-epimerase. J Immunol.

[7] Berry D, Lynn DM, Berry E, Sasisekharan R, Langer R (2006). Heparin localization and fine structure regulate Burkitt's lymphoma growth. Biochem Biophys Res Commun.

[8] Brühl H, Cihak J, Goebel N, Talke Y, Renner K, Hermann F, Rodriguez-Gomez M, Reich B, Plachý J, Stangassinger M, Mack M (2014). Chondroitin sulfate activates B cells *in vitro*, expands CD138+ cells *in vivo*, and interferes with established humoral immune responses. J Leukoc Biol.

[9] Korpetinou A, Skandalis SS, Labropoulou VT, Smirlaki G, Noulas A, Karamanos NK, Theocharis AD (2014). Serglycin: at the crossroad of inflammation and malignancy. Front Oncol.

[10] Kolset SO, Pejler G (2011). Serglycin: a structural and functional chameleon with wide impact on immune cells. J Immunol.

[11] Fadnes B, Husebekk A, Svineng G, Rekdal Ø, Yanagishita M, Kolset SO, Uhlin-Hansen L (2012). The proteoglycan repertoire of lymphoid cells. Glycoconj J.

[12] Anastasiadou E, Vaeth S, Cuomo L, Boccellato F, Vincenti S, Cirone M, Presutti C, Junker S, Winberg G, Frati L, Wade PA, Faggioni A, Trivedi P (2009). Epstein-Barr virus infection leads to partial phenotypic reversion of terminally differentiated malignant B cells. Cancer Lett.

[13] Carbone A, Gloghini A, Gaidano G, Franceschi S, Capello D, Drexler HG, Falini B, Dalla-Favera R (1998). Expression status of BCL-6 and syndecan-1 identifies distinct histogenetic subtypes of Hodgkin's disease. Blood.

[14] Sebestyén A, Kovalszky I, Mihalik R, Gallai M, Bocsi J, László E, Benedek S, Sréter L, Kopper L (1997). Expression of syndecan-1 in human B cell chronic lymphocytic leukaemia. Eur J Cancer.

[15] Kopper L, Sebestyén A, Gallai M, Kovalszky I (1997). Syndecan-1 - A new piece in B-cell puzzle. Pathol Oncol Res.

[16] Sebestyén A, Berczi L, Mihalik R, Paku S, Matolcsy A, Kopper L (1999). Syndecan-1 (CD138) expression in human non-Hodgkin lymphomas. Br J Haematol.

[17] Sebestyén A, Tótth A, Mihalik R, Szakács O, Paku S, Kopper L (2000). Syndecan-1-dependent homotypic cell adhesion in HT58 lymphoma cells. Tumour Biol.

[18] Spillmann D (2001). Heparan sulfate: anchor for viral intruders?. Biochimie.

[19] Liu J, Thorp SC (2002). Cell surface heparan sulfate and its roles in assisting viral infections. Med Res Rev.

[20] Zhu W, Li J, Liang G (2011). How does cellular heparan sulfate function in viral pathogenicity?. Biomed Environ Sci.

[21] Tan CW, Poh CL, Sam IC, Chan YF (2013). Enterovirus 71 uses cell surface heparan sulfate glycosaminoglycan as an attachment receptor. J Virol.

[22] Connell BJ, Lortat-Jacob H (2013). Human immunodeficiency virus and heparan sulfate: from attachment to entry inhibition. Front Immunol.

[23] Plochmann K, Horn A, Gschmack E, Armbruster N, Krieg J, Wiktorowicz T, Weber C, Stirnnagel K, Lindemann D, Rethwilm A, Scheller C (2012). Heparan sulfate is an attachment factor for foamy virus entry. J Virol.

[24] Akula SM, Wang FZ, Vieira J, Chandran B (2001). Human herpesvirus 8 interaction with target cells involves heparan sulfate. Virology.

[25] Bacsa S, Karasneh G, Dosa S, Liu J, Valyi-Nagy T, Shukla D (2011). Syndecan-1 and syndecan-2 play key roles in herpes simplex virus type-1 infection. J Gen Virol.

[26] Antoine TE, Park PJ, Shukla D (2013). Glycoprotein targeted therapeutics: a new era of anti-herpes simplex virus-1 therapeutics. Rev Med Virol.

[27] Lun SW, Cheung ST, Cheung PF, To KF, Woo JK, Choy KW, Chow C, Cheung CC, Chung GT, Cheng AS, Ko CW, Tsao SW, Busson P, Ng MH, Lo KW (2012). CD44+ cancer stem-like cells in EBV-associated nasopharyngeal carcinoma. PLoS One.

[28] Janisiewicz AM, Shin JH, Murillo-Sauca O, Kwok S, Le QT, Kong C, Kaplan MJ, Sunwoo JB (2012). CD44(+) cells have cancer stem cell-like properties in nasopharyngeal carcinoma. Int Forum Allergy Rhinol.

[29] Groma V, Kazanceva A, Nora-Krukle Z, Murovska M (2012). Oropharyngeal malignant epithelial cell, lymphocyte and macrophage CD44 surface receptors for hyaluronate are expressed in sustained EBV infection: immunohistochemical data and EBV DNA tissue indices. Pathol Res Pract.

[30] Chong JM, Fukayama M, Hayashi Y, Funata N, Takizawa T, Koike M, Muraoka M, Kikuchi-Yanoshita R, Miyaki M, Mizuno S (1997). Expression of CD44 variants in gastric carcinoma with or without Epstein-Barr virus. Int J Cancer.

[31] Abed N, Casper JT, Camitta BM, Margolis D, Trost B, Orentas R, Chang CC (2004). Evaluation of histogenesis of B-lymphocytes in pediatric EBV-related post-transplant lymphoproliferative disorders. Bone Marrow Transplant.

[32] Halder S, Murakami M, Verma SC, Kumar P, Yi F, Robertson ES (2009). Early events associated with infection of Epstein-Barr virus infection of primary B-cells. PLoS One.

[33] Rincon J, Prieto J, Patarroyo M (1992). Expression of integrins and other adhesion molecules in Epstein-Barr virus-transformed B lymphoblastoid cells and Burkitt's lymphoma cells. Int J Cancer.

[34] Kryworuchko M, Gee K, Diaz-Mitoma F, Kumar A (1999). Regulation of CD44-hyaluronan interactions in Burkitt's lymphoma and Epstein-Barr virus-transformed lymphoblastoid B cells by PMA and interleukin-4. Cell Immunol.

[35] Walter J, Schirrmacher V, Mosier D (1995). Induction of CD44 expression by the Epstein-Barr virus latent membrane protein LMP1 is associated with lymphoma dissemination. Int J Cancer.

[36] Birkenbach M, Josefsen K, Yalamanchili R, Lenoir G, Kieff E (1993). Epstein-Barr virus-induced genes: first lymphocyte-specific G protein-coupled peptide receptors. J Virol.

[37] Karpova MB, Schoumans J, Blennow E, Ernberg I, Henter JI, Smirnov AF, Nordenskjöld M, Fadeel B (2006). Combined spectral karyotyping, comparative genomic hybridization, and *in vitro* apoptyping of a panel of Burkitt's lymphoma-derived B cell lines reveals an unexpected complexity of chromosomal aberrations and a recurrence of specific abnormalities in chemoresistant cell lines. Int J Oncol.

[38] Masucci MG, Contreras-Salazar B, Ragnar E, Falk K, Minarovits J, Ernberg I, Klein G (1989). 5-Azacytidine up regulates the expression of Epstein-Barr virus nuclear antigen 2 (EBNA-2) through EBNA-6 and latent membrane protein in the Burkitt's lymphoma line rael. J Virol.

[39] Sun C, Liu X, Chen Y, Liu F (2006). Anticancer activities of trichostatin A on maligant lymphoid cells. J Huazhong Univ Sci Technolog Med Sci.

[40] Tancred TM, Belch AR, Reiman T, Pilarski LM, Kirshner J (2009). Altered expression of fibronectin and collagens I and IV in multiple myeloma and monoclonal gammopathy of undetermined significance. J Histochem Cytochem.

[41] Daoussis D, Liossis SN (2013). B cells tell scleroderma fibroblasts to produce collagen. Arthritis Res Ther.

[42] François A, Chatelus E, Wachsmann D, Sibilia J, Bahram S, Alsaleh G, Gottenberg JE (2013). B lymphocytes and B-cell activating factor promote collagen and profibrotic markers expression by dermal fibroblasts in systemic sclerosis. Arthritis Res Ther.

[43] Nadav-Dagan L, Shay T, Dezorella N, Naparstek E, Domany E, Katz BZ, Geiger B (2010). Adhesive interactions regulate transcriptional diversity in malignant B cells. Mol Cancer Res.

[44] Cader FZ, Vockerodt M, Bose S, Nagy E, Brundler MA, Kearns P, Murray PG (2013). The EBV oncogene LMP1 protects lymphoma cells from cell death through the collagen-mediated activation of DDR1. Blood.

[45] Rowe M, Lear AL, Croom-Carter D, Davies AH, Rickinson AB (1992). Three pathways of Epstein-Barr virus gene activation from EBNA1-positive latency in B lymphocytes. J Virol.

[46] Ernberg I, Falk K, Minarovits J, Busson P, Tursz T, Masucci MG, Klein G (1989). The role of methylation in the phenotype-dependent modulation of Epstein-Barr nuclear antigen 2 and latent membrane protein genes in cells latently infected with Epstein-Barr virus. J Gen Virol.

[47] Falk KI, Szekely L, Aleman A, Ernberg I (1998). Specific methylation patterns in two control regions of Epstein-Barr virus latency: the LMP-1-coding upstream regulatory region and an origin of DNA replication (oriP). J Virol.

[48] Takacs M, Segesdi J, Banati F, Koroknai A, Wolf H, Niller HH, Minarovits J (2009). The importance of epigenetic alterations in the development of epstein-barr virus-related lymphomas. Mediterr J Hematol Infect Dis.

[49] Niller HH, Szenthe K, Minarovits J (2014). Epstein-Barr virus-host cell interactions: an epigenetic dialog?. Front Genet.

[50] Niller HH, Wolf H, Minarovits J (2009). Epigenetic dysregulation of the host cell genome in Epstein-Barr virus-associated neoplasia. Semin Cancer Biol.

[51] Ryan JL, Jones RJ, Kenney SC, Rivenbark AG, Tang W, Knight ER, Coleman WB, Gulley ML (2010). Epstein-Barr virus-specific methylation of human genes in gastric cancer cells. Infect Agent Cancer.

[52] Cancer Genome Atlas Research Network (2014). Comprehensive molecular characterization of gastric adenocarcinoma. Nature.

[53] Suhovskih AV, Tsidulko AY, Kutsenko OS, Kovner AV, Aidagulova SV, Ernberg I, Grigorieva EV (2014). Transcriptional Activity of Heparan Sulfate Biosynthetic Machinery is Specifically Impaired in Benign Prostate Hyperplasia and Prostate Cancer. Front Oncol.

[54] Gutzeit C, Nagy N, Gentile M, Lyberg K, Gumz J, Vallhov H, Puga I, Klein E, Gabrielsson S, Cerutti A, Scheynius A (2014). Exosomes derived from Burkitt's lymphoma cell lines induce proliferation, differentiation, and class-switch recombination in B cells. J Immunol.

[55] Suhovskih AV, Aidagulova SV, Kashuba VI, Grigorieva EV (2015). Proteoglycans as potential microenvironmental biomarkers for colon cancer. Cell Tissue Res.

