# Supplementary material for: Proteoglycan expression correlates with the phenotype of malignant and non-malignant EBV-positive B-cell lines
Source: Oncotarget. 2015 Oct 16;6(41):43529–39. doi: 10.18632/oncotarget.5984 (PMC4791248; doi:10.18632/oncotarget.5984)
Supplement: Supplementary file 1 [file oncotarget-06-43529-s001.pdf]

SUPPLEMENTARY TABLE

Supplementary Table S1: Genes expression levels in normal B cells and different EBV+/- normal and cancer lymphoid cells

Genecell line	SDC1	GPC1	HSPG2	VCAN	BCAN	ACAN	NG2	SRGN	DCN	BGN	LUM	CD44
**B cells donor 1**	39,3 ± 30,1	66,4 ± 38,0	25,9 ± 7,8	3,7 ± 1,7	4,5 ± 3,4	34,0 ± 15,6	50,4 ± 19,2	**269,8** ± 20,5	22,6 ± 4,3	60,8 ± 47,9	49,8 ± 7,8	**374,6** ± 76,9
**B cells donor 2**	7,4 ± 1,2	9,4 ± 1,6	58,4 ± 14,9	3,6 ± 0,5	2,4 ± 0,2	0,8 ± 0,2	21,3 ± 6,2	**309,7** ± 51,7	17,7 ± 0,2	11,1 ± 2,3	40,7 ± 6,1	**560,6** ± 12,0
**CMB1-Ral-STO**	3,6 ± 4,3	1,2 ± 1,1	**219,3** ± 13,0	7,9 ± 9,4	7,4 ± 9,4	4,7 ± 6,1	0,6 ± 0,6	**684,3** ± 28,7	0,14 ± 0,11	2,2 ± 2,3	0,9 ± 1,0	4,13 ± 2,8
**CBC-JK2-STO**	3,5 ± 1,5	1,5 ± 1,2	**448,8** ± 142,9	13,0 ± 6,2	9,1 ± 8,2	10,8 ± 14	0,61 ± 0,32	**447,4** ± 1,1	0,11 ± 0,03	2,9 ± 2,6	1,0 ± 0,4	27,7 ± 4,6
**Nad20**	4,9 ± 4,6	1,6 ± 1,3	**245,4** ± 22,6	7,8 ± 9,66	6,5 ± 8,3	2,5 ± 3,3	0,6 ± 0,7	**741,2** ± 16,6	0,06 ± 0,02	2,0 ± 2,1	1,1 ± 1,3	62,8 ± 40,7
**Raji**	1,29 ± 0,8	2,3 ± 2,1	**296,2** ± 9,4	13,5 ± 6,8	5,4 ± 3,9	4,8 ± 4,8	0,4 ± 0,2	37,2 ± 22,2	0,08 ± 0,04	1,9 ± 1,4	0,7 ± 0,6	0,5 ± 0,3
**Daudi**	2,1 ± 1,5	2,8 ± 1,5	**376,7** ± 31,2	7,3 ± 6,2	8,8 ± 7,8	2,3 ± 3,1	0,96 ± 0,53	4,9 ± 0,1	0,1 ± 0,08	4,3 ± 4,3	1,3 ± 0,9	1,6 ± 0,8
**Mutu III cl99**	0,5 ± 0,6	0,7 ± 0,7	53,8 ± 25,5	1,3 ± 1,0	0,4 ± 0,2	0,2 ± 0,1	0,18 ± 0,07	49,3 ± 4,5	0,06 ± 0,04	1,2 ± 1,0	0,2 ± 0,1	0,4 ± 0,2
**Rael**	0,07 ± 0,08	1,2 ± 1,1	27,6 ± 3,7	0,2 ± 0,2	0,14 ± 0,06	0,007 ± 0,008	0,09 ± 0,06	6,2 ± 4,9	0,03 ± 0,04	0,23 ± 0,22	0,1 ± 0,04	0,11 ± 0,02
**Akata**	1,2 ± 0,1	0,9 ± 0,1	96,0 ± 28,0	5,0 ± 1,1	5,2 ± 4,9	1,1 ± 1,1	1,0 ± 1,1	5,8 ± 1,9	0,06 ± 0,07	2,2 ± 1,0	1,3 ± 0,4	1,06 ± 0,07
**Mutu I cl 148**	0,02 ± 0,02	0,7 ± 0,4	0,31 ± 0,07	0,02 ± 0,02	0,07 ± 0,03	0,1 ± 0,2	0,07 ± 0,01	0,910 ± ,03	0,02 ± 0,02	0,06 ± 0,01	0,03 ± 0,02	0,07 ± 0,03
**DG75**	8,0 ± 0,3	13,2 ± 9,3	4,5 ± 0,5	0,54 ± 0,05	1,3 ± 0,6	9,2 ± 5,8	7,2 ± 0,5	33,2 ± 0,6	3,6 ± 1,0	11,5 ± 7,9	11,2 ± 7,9	6,1 ± 1,9
